# Teosinte-Derived Advanced Backcross Population Harbors Genomic Regions for Grain Yield Attributing Traits in Maize

**DOI:** 10.3390/ijms251910300

**Published:** 2024-09-25

**Authors:** Pardeep Kumar, Mukesh Choudhary, Seema Sheoran, Ningthai Longmei, Bhupender Kumar, Bahadur Singh Jat, Manesh Chander Dagla, Bharat Bhushan, Sumit Kumar Aggarwal, Pravin Kumar Bagaria, Ankush Sharma, Shyam Bir Singh

**Affiliations:** 1 ICAR-Indian Institute of Maize Research, Ludhiana 141004, India; pardeepkumar656@gmail.com (P.K.); puilurongmei@gmail.com (N.L.); bhupender.iimr@yahoo.com (B.K.); bahadursinghnanda@gmail.com (B.S.J.); manu9322gen@gmail.com (M.C.D.); budding.biochemist@gmail.com (B.B.); sumit.aggarwal009@gmail.com (S.K.A.); pravin87hau@gmail.com (P.K.B.); imankushh@gmail.com (A.S.); singhsb1971@gmail.com (S.B.S.); 2 ICAR-Indian Agricultural Research Institute Regional Station, Karnal 132001, India; sheoranseema9@gmail.com

**Keywords:** teosinte, QTLs mapping, population, AB-QTL, wild

## Abstract

Maize is a highly versatile crop holding significant importance in global food, feed and nutritional security. Grain yield is a complex trait and difficult to improve without targeting the improvement of grain yield attributing traits, which are relatively less complex in nature. Hence, considering the erosion in genetic diversity, there is an urgent need to use wild relatives for genetic diversification and unravel the genomic regions for grain yield attributing traits in maize. Thus, the current study aimed to identify quantitative trait loci (QTLs) linked with grain yield and yield attributing traits. Two BC_2_F_2_ populations developed from the cross of LM13 with *Zea parviglumis* (population 1) and LM14 with *Zea parviglumis* (population 2) were genotyped and phenotyped in field conditions in the kharif season. BC_2_F_2:3_ lines in both populations were phenotyped again for grain yield and attributing traits in the spring season. In total, three QTLs each for ear height (EH), two QTLs for flag leaf length (FLL) and one QTL each for ear diameter (ED), plant height, flag leaf length (FLL), flag leaf width and 100 kernel-weight were identified in population 1. In population 2, two QTLs for kernel row per ear (KRPE) and one QTL for FLL were detected in. QTLs for EH, FLL and KPRE showed consistency across seasons. Among the identified QTLs, six QTLs were found to be co-localized near identified genomic regions in previous studies, validating their potential in contributing to trait expression. The identified QTLs can be utilized for marker assisted selection, transferring favorable alleles from wild relatives in modern maize.

## 1. Introduction

Maize is one of the most important and widely adapted cereal crops after rice and wheat in the world and the main source of food, feed, fodder, starch, biofuel and industrial applications [1,2,3]. Consequently, due to its diverse uses, the maize demand will further increase in the future. Therefore, to meet the demand in a changing climate scenario, improving yield under diverse ecologies should be the focus of maize researchers [4,5]. Yield is a complex trait and needs continuous breeding efforts for its improvement. Yield is an outcome of contribution of numerous yield attributing traits; hence, to optimize the yield expression, genetic manipulation of yield attributing traits is an effective way to enhance the yield [6].

Genetic diversity in the breeding germplasm is the key to improvement of grain yield and resistance/tolerance against biotic and abiotic stresses. Landraces, unadapted germplasm and wild species have enormous untapped genetic diversity for such traits and hence should be regularly utilized for genetic diversification in maize breeding programs [7]. Teosinte, commonly known as *Z. mays* ssp. *parviglumis*, is one of the progenitors of cultivated maize and possesses huge genetic diversity [1,8]. Pasztor and Borsos (1990) [9] and Srinivasan and Brewbaker (1999) [10] reported the genetic variability in teosinte for grain yield, yield attributing traits, disease resistance and insect pest tolerance.

The polygenic nature of yield and its associated traits necessitates its genetic dissection through quantitative trait loci (QTLs) analysis for subsequent use of QTLs in marker assisted selection. Tanksley and Nelson [11] introduced the Advanced Backcross Quantitative Trait Locus (AB-QTL) analysis in 1996 for identifying and transferring beneficial alleles from donor lines (wild or unadapted germplasm) into the elite cultivars. This technique allows for rapid development of improved lines that possess genomes nearly identical to the elite recurrent parent, as well as near isogenic lines containing the desired QTLs. The resulting improved lines can then be effectively used as donors or parents in maize breeding [12].

AB-QTL analysis has been successfully used in various crops like tomato, rice, maize, cotton, wheat and barley for identification of QTLs and transfer of favorable alleles from wild species into elite lines [13,14,15]. Hence, the present study aimed to utilize *Z. parviglumis* to develop two different BC_2_F_2_ and BC_2_F_2:3_ mapping populations, their evaluation in different seasons/environments and identification of genomic regions for grain yield and yield attributing traits. This study is expected to establish a foundation of utilization of pre-breeding populations in Indian maize breeding programs for genetic dissection of grain yield and component traits.

## 2. Results

### 2.1. Evaluation of BC_2_F_2_ Population

**Figure 1:**
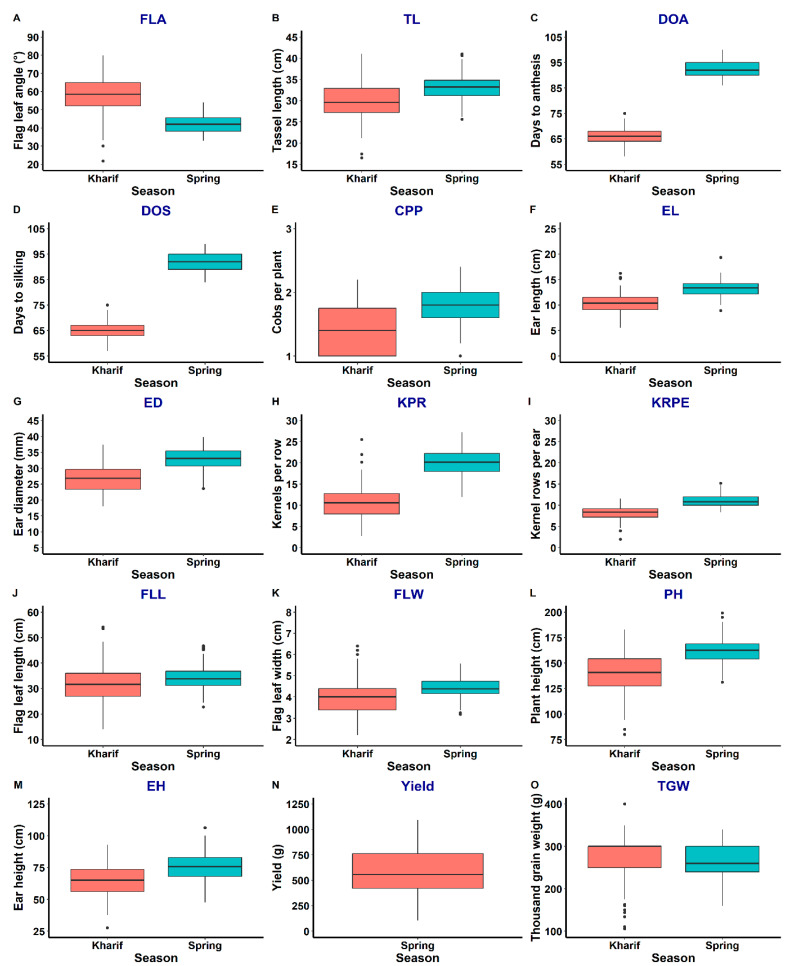
Box plot of variation for the traits under study in population 1 for kharif and spring seasons.

**Figure 2:**
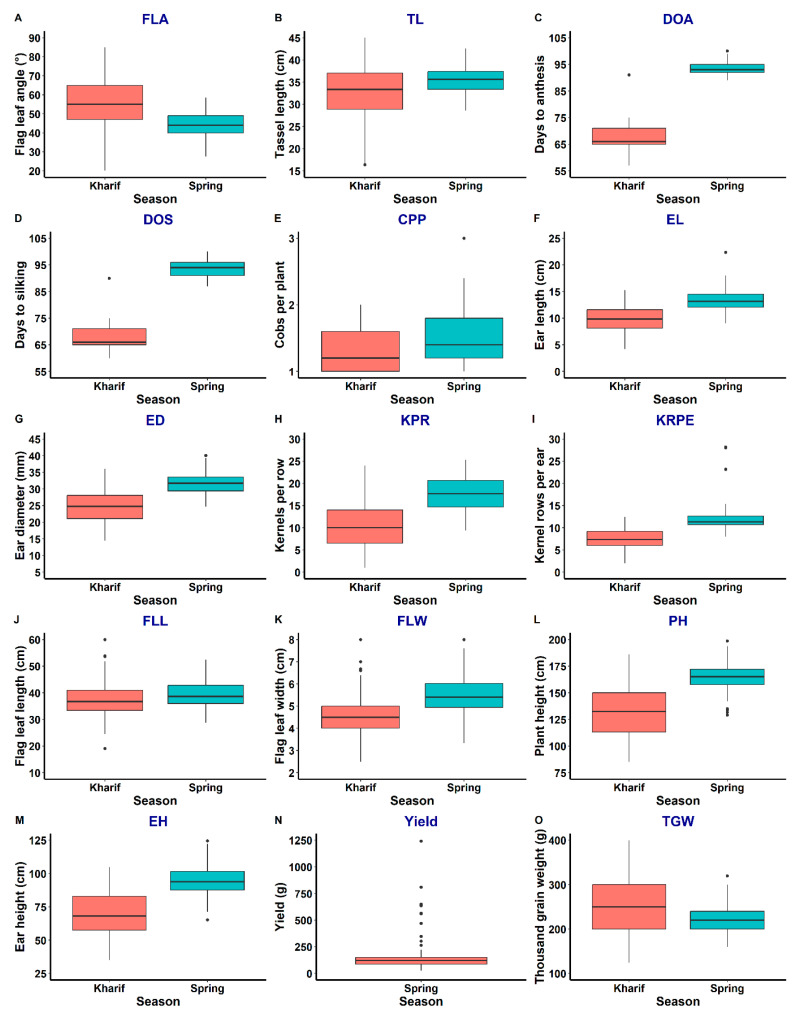
Box plot of variation for the traits under study in population 2 for kharif and spring seasons.

Traits measured in both populations (population 1 and population 2) showed different ranges of variation under the normal environment (natural conditions) in the kharif and spring seasons (Figure 1, Figure 2 and Figure S1). Spring has relatively lower temperatures during early growth (expanding the duration of spring crops); however, in later growth stages, the environmental factors are more or less similar to kharif. Population 1 showed a relatively greater range of variation for traits such as FLA, TL, CPP, ED, FLL, FLW and PH in kharif than spring. Similarly, population 2 showed greater variation for FLA, TL, DTA, EL, ED, KPR, PH, EH and 100 kW in kharif than spring. For GY, population 1 showed a larger range of variation in comparison to GY in population 2 in spring. A total of 57 significant correlations were detected in population 1 in kharif (Supplementary Materials Table S1). TL showed consistently significant and highly positive correlations with traits EL, ED, KPR, KRPE, FLL, FLW, PH, EH and 100 kW, whereas DTA showed positive correlation with DTS and significant negative correlation with CPP, EL, KPR, KRPE and FLW. Similarly, the trait EL had significant positive correlations with ED, KPR, KRPE, FLL, FLW, PH, EH and 100 kW. The associations of PH were significantly positive with almost all the traits except FLA, DTA and DTS. Similarly, 100 kW had significant and positive associations with TL, EL, ED, KPR, KRPE, PH and EH under kharif environments. In contrast, a total of 44 significant associations were observed for population 1 during spring (Supplementary Materials Table S2). GY showed significant positive correlation with EL, ED, KPR, KRPE, PH, EH and 100 kW and negative correlation with traits DTA and DTS. The 100 kW showed consistently significant and highly positive correlations with TL, ED, KR, KRPE, FLL, FLW and GY, whereas DTA showed positive correlation with DTS, PH and EH and negative correlation with ED, KPR, KRPE and GY.

A total of 43 significant associations were observed in population 2 during kharif, of which TL showed positive correlation with EL, KPR, KRPE, FLL, PH and 100 kW (Supplementary Materials Table S3). DTA is significantly negatively correlated with ED, KPR, KRPE and FLW and positively correlated with only DTS. EL was found to be positively correlated with TL, ED, KPR, KRPE, FLL, FLW, PH, EH and 100 kW, while PH showed positive correlation with TL, EL, ED, KPR, KRPE, FLL, FLW and EH. The 100 kW was found to be positively correlated with TL, EL, ED, KPR and KRPE. In contrast, only 14 significant trait correlations were found in population 2 during spring (Supplementary Materials Table S4). GY was found to be positively correlated with EL, KRPE and 100 kW, while TL and DTA showed positive correlation with FLL and DTS, respectively. EL was significantly and positively correlated with ED, KPR and GY, while PH was positively correlated with only EH (Section 2.1).

### 2.2. QTL Identification

A total of 8 QTLs were identified to be significantly associated with six grain yield related traits in population 1, while three QTLs were identified in population 2 in this study. The identified QTLs were located on chromosomes 2, 4, 5, 9 and 10.

#### 2.2.1. QTLs Identified from LM 13 × *Z. parviglumis* Derived BC_2_F_2_ Families (Population 1)

**Figure 3:**
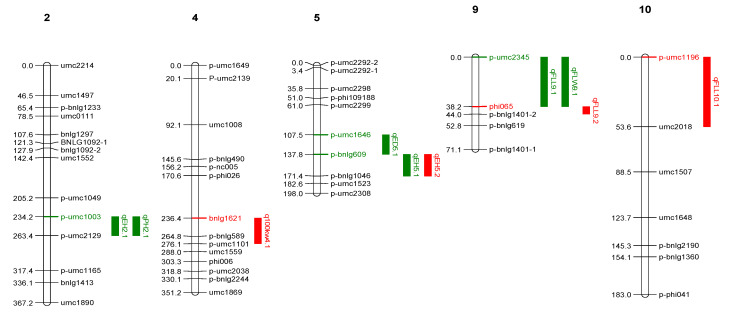
Linkage map with identified QTLs in population 1, green color (kharif season) and red color (spring season).

During kharif 2020, a total of six QTLs, including two QTLs each for ear height (*qEH2.1* and *qEH5.1*) and one each for ear diameter (*qED5.1*), plant height (*qPH2.1*), flag leaf length (*qFL9.1*) and flag leaf width (*qFLW9.1*), were identified in population 1 (Table 1, Figure 3). The LOD value and phenotypic coefficient of variation (PVE) for identified QTLs ranged from 3.82 to 6.40 and 11.50 to 17.60, respectively. Two QTLs for ear height (*qEH2.1* and *qEH5.1*) were located on chromosomes 2 and 5 with a PVE of 17.6 and 13.4 and LOD value of 6.4 and 4.74, respectively. Similarly, four QTLs were identified for three traits, namely 100 kernel weight (*q100kw4.1*), ear height (*qEH5.2*) and flag leaf length (*qFLL9.2* and *qFLL10.1*) on chromosomes 4, 5, 9 and 10 during spring 2021 in the same population. These QTLs had LOD and PVE values in the range of 3.68–4.82 and 10.80–13.90, respectively. Two QTLs, namely *qFLL9.1* for flag leaf length and *qEH5.1* for plant height, were consistently detected in both seasons.

#### 2.2.2. QTLs Identified from LM 14 × *Z. parviglumis* Derived BC_2_F_2_ Families (Population 2)

**Figure 4:**
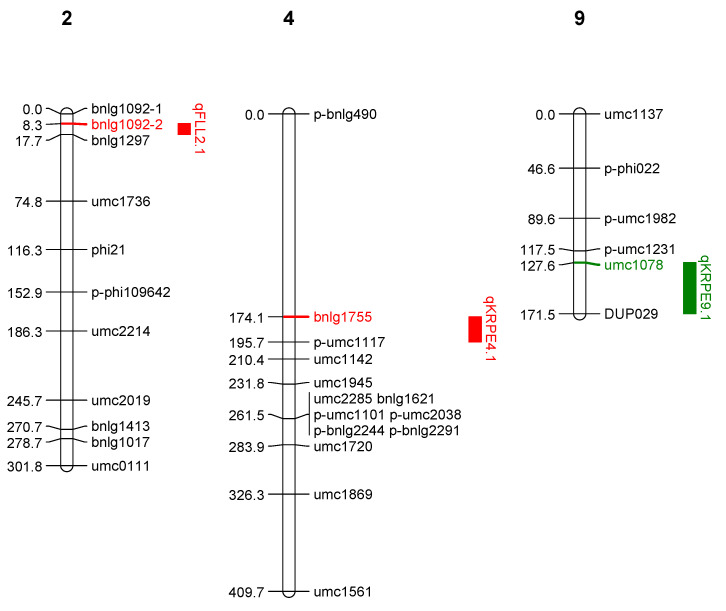
Linkage map with identified QTL in population 2, green color (kharif season) and red color (spring season).

Likewise, in population 2, one QTL (*qKRPE9.1*) for kernel rows per ear was mapped on chromosome 9, with a LOD of 3.70 and a PVE of 13.70 during kharif 2020 (Table 2, Figure 4). Similarly, two QTLs were identified for flag leaf length (*qFLL2.1*) and kernel rows per ear (*qKRPE4.1*) on chromosomes 2 and 4 during spring 2021. QTLs for flag leaf lengths i.e., *qFLL9.1* and *qFLL2.1*, were identified in both the populations but on different chromosomes, i.e., 9 and 2, respectively. Similarly, QTLs for kernel rows per ear, i.e., *qKRPE9.1* and *qKRPE4.1*, were detected in both seasons but on different chromosomes, i.e., 9 and 4, respectively.

## 3. Discussion

### 3.1. Comparison of QTLs Detected in Both BC_2_F_2_ Populations

In both population 1 and population 2, ten and three QTLs were detected, respectively, for the six grain yield related component traits. Out of a total of 13 QTLs, only one QTL for flag leaf length (accounting for ~11.5% PVE) was detected in both the populations but located on different chromosomes. This indicates the governance of flag leaf length by different clusters of genes located in different chromosomes, and such QTLs are of genotype specific nature as favorable alleles are contributed by *Z. parviglumis* and LM 14. Such genotype specific QTLs have been detected previously in maize based studies [5]. The remaining 12 QTLs were specific to population 1, and two QTLs were specific to population 2. Such population specific detection of QTLs is quite common as evident from previous studies [16]. In population 1, the QTLs for flag leaf length (*qFLL 9.1* and *qFLL9.2*) were detected in both the seasons that are located on chromosome 9 (p-umc2345 and phi065) and with a PVE of 12% and hence classify as consistent or stable QTLs. Such stable QTLs are of high interest to plant breeders, as leaves are a primary source for photosynthesis for overall plant growth [5]. Similarly, the QTLs for ear height (*qEH 5.1* and *qEH 5.2*) were also identified as stable QTLs and hold significant importance for the development of genotypes with low ear placement, best suited for high planting density [17]. Interestingly, in population 1 evaluated in kharif 2020, QTLs for EH (*qEH2.1*) and PH (*qPH2.1*) were found to be co-located with p-umc2129 as the flanking common marker on chromosome 2, indicating the pleiotropic or correlated nature of such overlapping QTL regions. Detection of such consistent QTLs indicates relatively higher heritability of these traits, indicating a low effect of environment on expression. The trait correlation analysis indicated significant positive correlation between PH and EH in population 1 (Supplementary Materials Table S1) and corroborates with the findings of Fei et al., 2022 [18].

Likewise, QTLs for ED (*qED5.1*) and EH (*qEH5.1*) on chromosome 5 also overlapped and had p-bnlg609 as a common flanking marker and hence can be considered pleiotropic loci. The trait correlation analysis showed significant positive association between ED and EH and agrees with previous findings [19]. Similarly, QTLs for FLL (*qFLL9.1*) and FLW (*qFLW9.1*) on chromosome 9 overlapped due to significant positive correlation between FLL and FLW (Supplementary Materials Table S1). High positive correlations of traits are quite common for the co-located QTLs [20]. The highest number of QTLs were identified in population 1, possibly due to relatively larger variation captured for the phenotypic traits (Figure 4). Most of the QTLs identified in the present study are novel, possibly due to limited studies on the use of wild species for genetic mapping of grain yield and component traits. QTL detection inconsistency across two populations (where one of the parents is common) results from different genetic backgrounds, environmental factors or the selection process. However, in the current study, both BC_2_F_2_ populations were evaluated in the same location in two environments/seasons, and an almost similar set of polymorphic markers was used for genotyping both populations. Moreno-Gonzalez (1993) [21] demonstrated through a simulation study that the efficiency of different generations in predicting marker-associated QTL effects by multiple regression varies. Beavis et al. (1994) [22] proposed that variation in QTL detection between two F_3_ derived backcrossed lines might be attributed, in part, to the genetic background. The identified QTLs can be used in the genomic prediction models for grain yield contributing traits, resulting in better selection accuracy and achieving higher genetic gains for grain yield.

### 3.2. Comparison of QTLs Detected in Both BC_2_F_2_ Populations and Other Studies in Maize

Because of differences in mapping populations (parents and progeny type), as well as a paucity of common loci and environments, direct comparisons of QTL mapping results across studies are difficult. One important consideration for QTL detection is the degree to which QTL location and effects from one population are observed in other populations or subsamples of the same population. Inconsistent detection of QTLs across studies may be the result of sampling variation, genetic heterogeneity of the phenotype and other factors [22,23,24]. Although there are some studies in maize where common QTLs in similar genomic regions or neighboring regions on the same chromosome across populations for various traits were reported [25,26], the comparison of QTL data across different studies in maize provides clues on shared genomic regions, unraveling the genetic background effects or environmental effect [27]. Such QTLs identified across studies can be used for meta-QTL analysis to identify overlapping but consistent genomic regions, which can further be explored to mine candidate genes for particular traits.

Similar to our study, Adhikari et al., 2021 [28] identified QTLs for ear diameter and kernel rows per ear in BC_1_F_5_ population derived from the cross of maize inbred line DI-103 with *Z. parviglumis*. Similarly, QTLs for ED were reported in F_2:3_, and RIL population in maize [29,30,31,32] carried out QTL analysis using F_2_ and F_2:3_ populations and detected three QTLs for ear length. Similarly, Sa et al. (2021) [33] used RIL population (cross of Mo17 and KW7) to identify a QTL for ear length on chromosome 6. Hence, it is a great challenge to identify consistent QTLs across studies in different genetic backgrounds and environments, making QTL validation a daunting task; however, meta-QTL analysis is a promising approach for utilizing the existing QTL mapping studies [18,34].

## 4. Materials and Methods

### 4.1. Population Development

**Figure 5:**
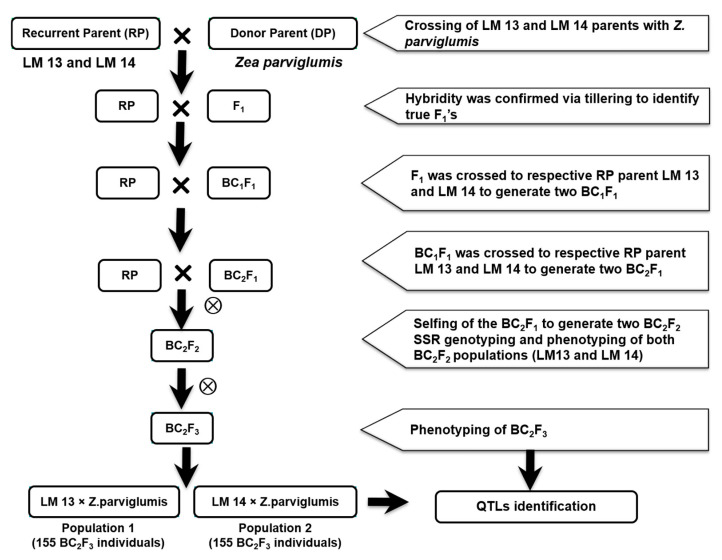
Flow chart for development of advanced backcross populations.

Two mapping populations were developed through the cross of maize inbred lines- LM 13 and LM 14 with *Z. parviglumis* (maize wild species). The F_1_ plants from each cross were backcrossed two times to LM13 and LM 14 to develop BC_2_F_1_ generation. BC_2_F_1_ plants were selfed to produce 155 BC_2_F_2_ individuals (population 1; based on LM 13) and 156 BC_2_F_2_ individuals (population 2; based on LM 14), respectively. Furthermore, BC_2_F_2_ plants were selfed to produce BC_2_F_3_ families in both populations (Figure 5).

### 4.2. Phenotypic Trait Evaluation

Both BC_2_F_2_ populations (population 1 and 2) were evaluated in one-row plots during kharif 2020, at Ladhowal farm of ICAR-IIMR, Ludhiana. Standard cultivation management practices were used. Both the populations were evaluated for 15 traits, viz., Flag Leaf Angle (FLA), Tassel Length (TL), Days to Anthesis (DTA), Days to Silking (DTS), Cobs per Plant (CPP), Ear Length (EL), Ear Diameter (ED), Kernels per row (KPR), Kernel rows per Ear (KRPE), Flag Leaf Length (FLL), Flag leaf width (FLW), Plant Height (PH), Ear Height (EH), Grain Yield (GY) and 100 kernel weight (100 kW). As the population is segregated, the phenotypic data were collected from individual plants in BC_2_F_2_, and yield attributing traits (EL, ED, KPR, KRPE, 100 kW) were recorded on selfed plant, as it was necessary to generate the BC_2_F_3_ families. BC_2_F_3_ populations (population 1 and population 2) were evaluated for above mentioned traits and grain yield (in open pollination conditions) during spring 2021 and the data were collected on five random plants.

### 4.3. Correlation and Box Plot Analysis

The correlation coefficients among the grain yield and its component traits were calculated, and box plots were generated using the agricolae package (ggplot2) of R software Version 4.4.1.

### 4.4. Linkage Map Construction and QTLs Mapping

A total of 180 and 162 SSR markers were used to screen populations 1 and 2, respectively. Based on the polymorphism, 102 and 77 markers were used to construct the linkage maps for population 1 (Figure 3, Supplementary Materials Table S5) and population 2 (Figure 4, Supplementary Materials Table S6), respectively. The linkage maps covered 10 chromosomes with a total length of 2524.8 cM and 2139.4 cM, with an average interval of 24.75 cM and 27.78 cM, respectively. The linkage map was constructed using ICI QTL mapping software v4.1. Further, QTLs were mapped using the QGENE 4.4.0 after selecting 1000 permutations. The negative additive effect indicates that the QTL allele was contributed from the donor parent while positive value indicates the QTL allele is from the recurrent parent.

## 5. Conclusions

This study resulted in the development of pre-breeding populations, linkage map construction and identification of genomic regions for grain yield and yield attributing traits. The co-localization of QTLs (with close and common flanking markers) for yield attributing traits due to their strong associations provides the opportunity for their simultaneous improvement. Such co-located QTLs for same or different traits can be targeted for marked assisted introgression in elite maize germplasm. The identification of QTLs for yield-related traits conferred by wild relatives in the modern maize background will enrich genetic diversity in the germplasm. Furthermore, other QTLs identified for grain yield related traits were positioned from their linked markers, indicating the need for identifying close markers through fine mapping of such genomic regions. Moreover, such mapping populations in advanced generation (RILs) can be further evaluated to validate the identified QTLs.

## Figures and Tables

**Table 1:** QTLs reported from BC_2_F_2_ (LM 13 × *Z. parviglumis*) population for yield and contributing traits.

S. No.	Traits	Chr.	QTLs	LOD	R^2^ (%)	Linked Markers	Add. Effect
	Kharif 2020						
1	Ear Diameter	5	*qED5.1*	3.82	12.10	p-umc1646 & p-bnlg609	3.44
2	Ear Height	2	*qEH2.1*	6.40	17.60	p-umc1003 & p-umc2129	−12.45
		5	*qEH5.1*	4.74	13.40	p-bnlg609 & p-bnlg1046	19.17
3	Plant Height	2	*qPH2.1*	4.30	12.20	p-umc1049 & p-umc2129	−17.49
4	Flag leaf length	9	*qFLL9.1*	4.38	12.40	p-umc2345 & phi065	−9.56
5	Flag leaf width	9	*qFLW9.1*	4.05	11.50	p-umc2345 & phi065	−0.33
Spring 2021							
6	100 kernel weight	4	*q100kw4.1*	4.48	12.90	bnlg1621 & p-umc1101	1.77
7	Ear height	5	*qEH5.2*	4.82	13.90	p-umc609 & p-bnlg1046	8.62
8	Flag leaf length	9	*qFLL9.2*	4.13	12.00	phi065 & p-bnlg1401-2	10.92
		10	*qFLL10.1*	3.68	10.80	umc1196 & umc2018	−3.00

**Table 2:** QTLs reported from BC_2_F_2_ (LM 14 × *Z. parviglumis*) population for yield and contributing traits.

S. No.	Traits	Chr.	QTLs	LOD	R^2^ (%)	Linked Markers	Add. Effect
	Kharif 2020						
1	Kernel rows per ear	9	*qKRPE9.1*	3.70	13.70	umc1078 & DUP029	−8.33
Spring 2021							
2	Flag leaf length	2	*qFLL2.1*	3.64	10.30	bnlg1092-2 & bnlg1297	2.61
3	Kernel rows per ear	4	*qKRPE4.1*	5.09	14.60	bnlg1755 & pumc1117	7.80

## Data Availability

Data are contained within the article and Supplementary Materials.

## Supplementary Materials

The supporting information can be downloaded at: https://www.mdpi.com/article/10.3390/ijms251910300/s1.
