# Supplementary material for: Teosinte-Derived Advanced Backcross Population Harbors Genomic Regions for Grain Yield Attributing Traits in Maize
Source: Int J Mol Sci. 2024 Sep 25;25(19):10300. doi: 10.3390/ijms251910300 (PMC11476406; doi:10.3390/ijms251910300)
Supplement: Supplementary file 1 [file ijms-25-10300-s001.zip › ijms-3217456-supplementary.pdf]

Supplementary Table S1: Correlation among the yield and yield contributing traits in Population 1_Kharif season

	FLA	TL	DTA	DTS	CPP	EL	ED	KPE	KRPE	FLL	FLW	PH	EH	1000kw
FLA	1	0.032	0.115	0.101	0.026	0.014	-0.031	-0.058	0.02	0.097	0.14	-0.047	-0.002	0.008
TL	0.032	1	-0.114	-0.101	0.139	0.52***	0.349***	0.418***	0.32***	0.384***	0.279***	0.485***	0.458***	0.289***
DTA	0.115	-0.114	1	0.968***	-0.214**	-0.336***	-0.134	-0.324***	-0.302***	-0.122	-0.317***	-0.137	-0.137	-0.108
DTS	0.101	-0.101	0.968***	1	-0.188*	-0.35***	-0.102	-0.314***	-0.291***	-0.142	-0.323***	-0.153	-0.147	-0.096
CPP	0.026	0.139	-0.214**	-0.188*	1	0.132	0.14	0.075	0.223**	0.207*	0.373***	0.285***	0.215**	0.146
EL	0.014	0.52***	-0.336***	-0.35***	0.132	1	0.591***	0.818***	0.625***	0.328***	0.323***	0.458***	0.447***	0.39***
ED	-0.031	0.349***	-0.134	-0.102	0.14	0.591***	1	0.722***	0.74***	0.213*	0.146	0.357***	0.357***	0.519***
KPE	-0.058	0.418***	-0.324***	-0.314***	0.075	0.818***	0.722***	1	0.772***	0.219**	0.192*	0.435***	0.419***	0.437***
KRPE	0.02	0.32***	-0.302***	-0.291***	0.223**	0.625***	0.74***	0.772***	1	0.13	0.169*	0.394***	0.364***	0.505***
FLL	0.097	0.384***	-0.122	-0.142	0.207*	0.328***	0.213*	0.219**	0.13	1	0.69***	0.328***	0.363***	0.007
FLW	0.14	0.279***	-0.317***	-0.323***	0.373***	0.323***	0.146	0.192*	0.169*	0.69***	1	0.383***	0.374***	0.098
PH	-0.047	0.485***	-0.137	-0.153	0.285***	0.458***	0.357***	0.435***	0.394***	0.328***	0.383***	1	0.85***	0.178*
EH	-0.002	0.458***	-0.137	-0.147	0.215**	0.447***	0.357***	0.419***	0.364***	0.363***	0.374***	0.85***	1	0.187*
100kw	0.008	0.289***	-0.108	-0.096	0.146	0.39***	0.519***	0.437***	0.505***	0.007	0.098	0.178*	0.187*	1

*** Correlation is significant at 0.001 level (two tailed) ** Correlation is significant at 0.01 level (two tailed) * Correlation is significant at 0.05 level (two tailed)

Flag Leaf Angle (FLA), Tassel Length (TL), Days to Anthesis (DTA), Days to Silking (DTS), Cobs per Plant (CPP), Ear Length (EL), Ear Diameter (ED), Kernels per row (KPR), Kernel rows per Ear (KRPE), Flag Leaf Length (FLL), Flag leaf width (FLW), Plant Height (PH), Ear Height (EH), Grain Yield (GY) and 100 kernel weight (100kw).

Supplementary Table S2: Correlation among the yield and yield contributing traits in Population 1_Spring season

	FLA	TL	DTA	DTS	CPP	EL	ED	KPR	KRPE	FLL	FLW	PH	EH	1000kw	GY
FLA	1	-0.062	0.1	0.1	-0.018	0.014	-0.065	-0.085	-0.103	0.129	0.186*	-0.117	0.012	-0.063	-0.121
TL	-0.062	1	-0.114	-0.155	0.111	0.162*	0.117	0.095	0.04	0.454***	0.193*	0.124	0.024	0.177*	0.014
DTA	0.1	-0.114	1	0.873***	0.051	-0.044	-0.315***	-0.207*	-0.379***	-0.004	-0.119	0.243**	0.183*	-0.137	-0.244**
DTS	0.1	-0.155	0.873***	1	0.038	-0.079	-0.298***	-0.223**	-0.371***	0.002	-0.111	0.19*	0.107	-0.177*	-0.3***
CPP	-0.018	0.111	0.051	0.038	1	-0.012	0.034	-0.015	-0.035	0.03	-0.119	0.063	0.167*	-0.022	0.08
EL	0.014	0.162*	-0.044	-0.079	-0.012	1	0.518***	0.773***	0.501***	0.168*	-0.027	0.18*	0.293***	0.134	0.282***
ED	-0.065	0.117	-0.315***	-0.298***	0.034	0.518***	1	0.582***	0.788***	0.073	0.115	0.216**	0.345***	0.406***	0.549***
KPR	-0.085	0.095	-0.207*	-0.223**	-0.015	0.773***	0.582***	1	0.595***	0.05	-0.098	0.203*	0.334***	0.111	0.502***
KRPE	-0.103	0.04	-0.379***	-0.371***	-0.035	0.501***	0.788***	0.595***	1	0.072	0.106	0.141	0.163*	0.304***	0.427***
FLL	0.129	0.454***	-0.004	0.002	0.03	0.168*	0.073	0.05	0.072	1	0.36***	0.061	-0.046	0.31***	0.005
FLW	0.186*	0.193*	-0.119	-0.111	-0.119	-0.027	0.115	-0.098	0.106	0.36***	1	0.028	0.044	0.218**	0.012
PH	-0.117	0.124	0.243**	0.19*	0.063	0.18*	0.216**	0.203*	0.141	0.061	0.028	1	0.36***	0.055	0.225**
EH	0.012	0.024	0.183*	0.107	0.167*	0.293***	0.345***	0.334***	0.163*	-0.046	0.044	0.36***	1	0.077	0.323***
100kw	-0.063	0.177*	-0.137	-0.177*	-0.022	0.134	0.406***	0.111	0.304***	0.31***	0.218**	0.055	0.077	1	0.384***
GY	-0.121	0.014	-0.244**	-0.3***	0.08	0.282***	0.549***	0.502***	0.427***	0.005	0.012	0.225**	0.323***	0.384***	1

*** Correlation is significant at 0.001 level (two tailed) ** Correlation is significant at 0.01 level (two tailed) * Correlation is significant at 0.05 level (two tailed)

Supplementary Table S3: Correlation among the yield and yield contributing traits in Population 2_Kharif season

	FLA	TL	DTA	DTS	CPP	EL	ED	KPR	KRPE	FLL	FLW	PH	EH	1000kw
FLA	1	-0.217*	-0.089	-0.095	-0.013	-0.086	-0.086	-0.115	-0.109	-0.106	0.029	-0.014	0.088	-0.106
TL	-0.217*	1	0.068	0.087	0.129	0.513***	0.164	0.373***	0.215*	0.455***	0.063	0.34***	0.074	0.282**
DTA	-0.089	0.068	1	0.981***	-0.048	-0.127	-0.232*	-0.255**	-0.265**	-0.094	-0.215*	-0.009	-0.028	-0.157
DTS	-0.095	0.087	0.981***	1	-0.036	-0.093	-0.209*	-0.216*	-0.247**	-0.086	-0.185*	0.017	0.007	-0.15
CPP	-0.013	0.129	-0.048	-0.036	1	0.078	-0.025	0.078	-0.066	0.123	0.124	0.118	0.006	-0.032
EL	-0.086	0.513***	-0.127	-0.093	0.078	1	0.428***	0.665***	0.509***	0.374***	0.283**	0.469***	0.353***	0.458***
ED	-0.086	0.164	-0.232*	-0.209*	-0.025	0.428***	1	0.662***	0.823***	0.197*	0.174	0.262**	0.23*	0.409***
KPR	-0.115	0.373***	-0.255**	-0.216*	0.078	0.665***	0.662***	1	0.792***	0.219*	0.181	0.36***	0.314***	0.484***
KRPE	-0.109	0.215*	-0.265**	-0.247**	-0.066	0.509***	0.823***	0.792***	1	0.179	0.118	0.324***	0.307**	0.393***

	FLA	TL	DTA	DTS	CPP	EL	ED	KPR	KRPE	FLL	FLW	PH	EH	1000kw
FLL	-0.106	0.455***	-0.094	-0.086	0.123	0.374***	0.197*	0.219*	0.179	1	0.645***	0.276***	0.232**	0.032
FLW	0.029	0.063	-0.215*	-0.185*	0.124	0.283**	0.174	0.181	0.118	0.645***	1	0.201*	0.315***	-0.034
PH	-0.014	0.34***	-0.009	0.017	0.118	0.469***	0.262**	0.36***	0.324***	0.276***	0.201*	1	0.822***	0.145
EH	0.088	0.074	-0.028	0.007	0.006	0.353***	0.23*	0.314***	0.307**	0.232**	0.315***	0.822***	1	-0.014
100kw	-0.106	0.282**	-0.157	-0.15	-0.032	0.458***	0.409***	0.484***	0.393***	0.032	-0.034	0.145	-0.014	1

*** Correlation is significant at 0.001 level (two tailed) ** Correlation is significant at 0.01 level (two tailed) * Correlation is significant at 0.05 level (two tailed)

Supplementary Table S4: Correlation among the yield and yield contributing traits in Population 2_Spring season

	FLA	TL	DTA	DTS	CPP	EL	ED	KPR	KRPE	FLL	FLW	PH	EH	1000kw	GY
FLA	1	-0.064	-0.086	-0.105	-0.033	0.042	-0.033	-0.032	-0.065	0.104	0.16*	-0.157	-0.074	0.156	0.047
TL	-0.064	1	0.045	0.055	0.142	0.076	0.066	0.075	0.072	0.367***	0.026	0.049	-0.012	-0.074	0.012
DTA	-0.086	0.045	1	0.81***	0.126	0	0.129	-0.004	-0.047	-0.036	-0.135	-0.024	0.086	-0.005	-0.073
DTS	-0.105	0.055	0.81***	1	-0.012	0.139	0.13	0.03	-0.071	-0.02	-0.12	-0.045	0.097	0.029	0.023
CPP	-0.033	0.142	0.126	-0.012	1	0.111	-0.027	0.147	-0.031	-0.198*	-0.248**	-0.01	-0.091	-0.136	0.058
EL	0.042	0.076	0	0.139	0.111	1	0.272***	0.416***	-0.036	0.033	-0.07	-0.071	-0.024	0.083	0.223**
ED	-0.033	0.066	0.129	0.13	-0.027	0.272***	1	0.399***	0.084	0.018	0.095	0.031	0.041	0.24**	0.061
KPR	-0.032	0.075	-0.004	0.03	0.147	0.416***	0.399***	1	0.189*	-0.01	0.053	0.074	0.117	-0.145	0.053
KRPE	-0.065	0.072	-0.047	-0.071	-0.031	-0.036	0.084	0.189*	1	0.091	0.072	-0.041	0.06	0.089	0.483***
FLL	0.104	0.367***	-0.036	-0.02	-0.198*	0.033	0.018	-0.01	0.091	1	0.526***	-0.021	-0.036	0.045	0.001
FLW	0.16*	0.026	-0.135	-0.12	-0.248**	-0.07	0.095	0.053	0.072	0.526***	1	-0.019	0.087	0.099	-0.015
PH	-0.157	0.049	-0.024	-0.045	-0.01	-0.071	0.031	0.074	-0.041	-0.021	-0.019	1	0.682***	0.014	-0.05
EH	-0.074	-0.012	0.086	0.097	-0.091	-0.024	0.041	0.117	0.06	-0.036	0.087	0.682***	1	0.143	-0.014
100kw	0.156	-0.074	-0.005	0.029	-0.136	0.083	0.24**	-0.145	0.089	0.045	0.099	0.014	0.143	1	0.391***
GY	0.047	0.012	-0.073	0.023	0.058	0.223**	0.061	0.053	0.483***	0.001	-0.015	-0.05	-0.014	0.391***	1

*** Correlation is significant at 0.001 level (two tailed) ** Correlation is significant at 0.01 level (two tailed) * Correlation is significant at 0.05 level (two tailed)

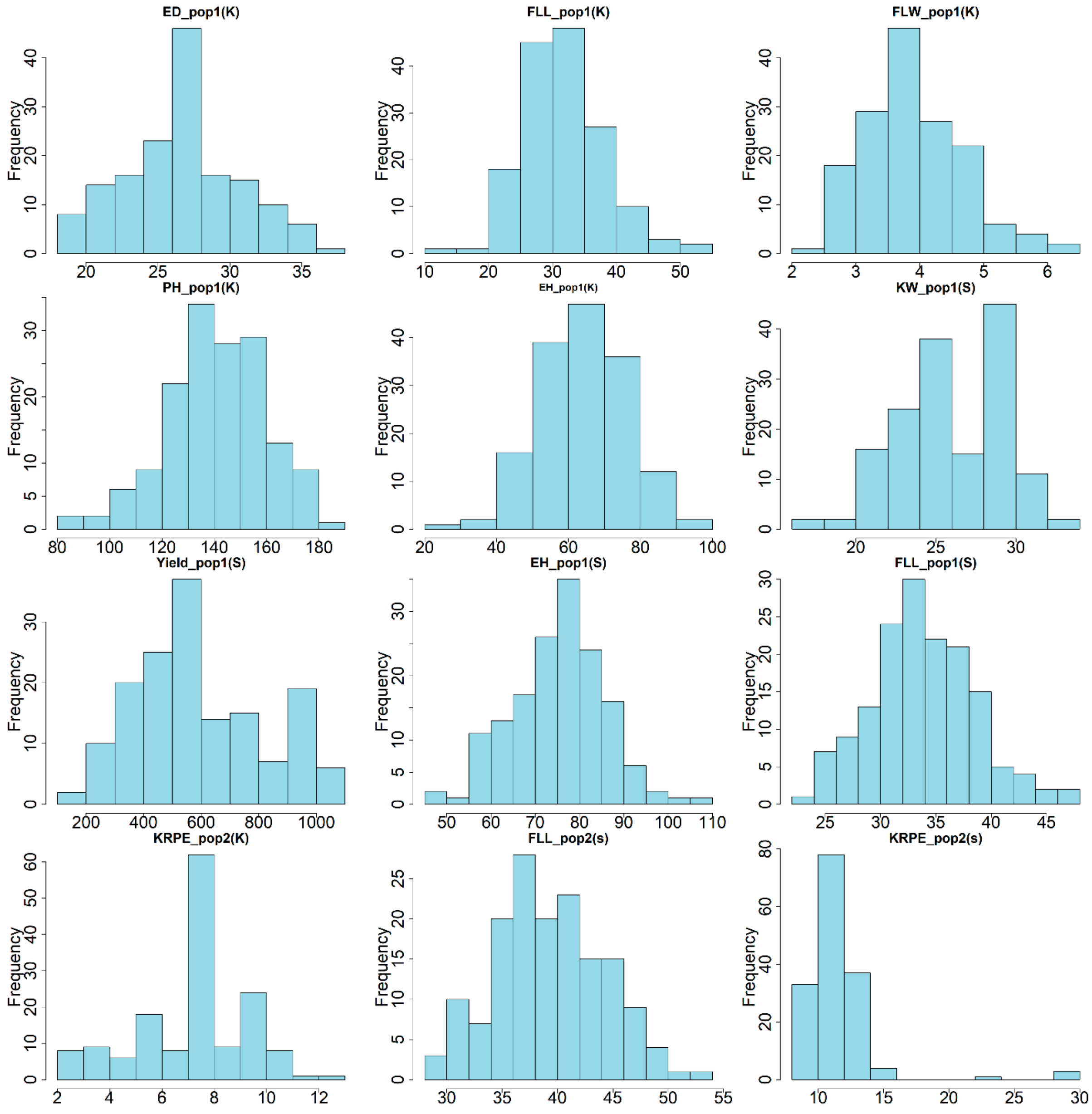

Supplementary Figure S1. Histogram for measured traits in population 1 and population 2 in kharif (K) and spring (S)

Supplementary Table S5: Position of different markers used in population 1

Chr.	position	locus	Chr.	position	locus	Chr.	position	locus	Chr.	position	locus	Chr.	position	locus
1	0.0	p-umc1568	2	0.0	umc2214	3	0.0	umc1102	4	0.0	p-umc1649	5	0.0	p-umc2292-2
1	22.1	p-umc1976	2	46.5	umc1497	3	89.2	umc1780	4	20.1	P-umc2139	5	3.4	p-umc2292-1
1	60.3	p-umc2012	2	65.4	p-bnlg1233	3	123.1	umc1813	4	92.1	umc1008	5	35.8	p-umc2298
1	77.3	umc2225	2	78.5	umc0111	3	175.4	bnlg1113	4	145.6	p-bnlg490	5	51.0	p-phi109188
1	82.9	bnlg1866	2	107.6	bnlg1297	3	247.3	p-umc1458	4	156.2	p-nc005	5	61.0	p-umc2299
1	117.7	umc0031	2	121.3	BNLG1092-1	3	264.4	p-bnlg1447	4	170.6	p-phi026	5	107.5	p-umc1646
1	145.3	p-umc2025-2	2	127.9	bnlg1092-2	3	302.5	p-umc1174	4	236.4	bnlg1621	5	137.8	p-bnlg609
1	169.5	umc2149	2	142.4	umc1552	3	350.5	umc2048	4	264.8	p-bnlg589	5	171.4	p-bnlg1046
1	184.4	p-umc2149	2	205.2	p-umc1049	3	370.6	p-umc2050	4	276.1	p-umc1101	5	182.6	p-umc1523
1	227.1	phi056	2	234.2	p-umc1003	3	390.6	umc-1659	4	288.0	umc1559	5	198.0	p-umc2308
1	276.2	p-umc2025-1	2	263.4	p-umc2129				4	303.3	phi006			
1	312.6	p-umc-1515	2	317.4	p-umc1165				4	318.8	p-umc2038			
1	330.4	p-bnlg1556-2	2	336.1	bnlg1413				4	330.1	p-bnlg2244			
1	348.9	p-bnlg1556-1	2	367.2	umc1890				4	351.2	umc1869			
1	363.7	UMC1297-1							4	394.7	phi213984			
1	401.5	umc1297-2												
**Chr.**	**position**	**locus**	**Chr.**	**position**	**locus**	**Chr.**	**position**	**locus**	**Chr.**	**position**	**locus**	**Chr.**	**position**	**locus**
6	0.0	umc2310	7	0.0	p-umc2333-2	8	0.0	p-bnlg162	9	0.0	p-umc2345	10	0.0	p-umc1196
6	21.5	p-umc2006	7	19.5	p-umc1295-1	8	34.1	umc1359	9	38.2	phi065	10	53.6	umc2018
6	33.7	bnlg2191	7	21.2	p-phi328175	8	49.6	P-BNLG1194	9	44.0	p-bnlg1401-2	10	88.5	umc1507
6	46.4	bnlg1600	7	36.9	p-umc1295-2	8	66.9	p-bnlg2235	9	52.8	p-bnlg619	10	123.7	umc1648
6	52.9	phi077	7	81.4	p-umc2177	8	87.5	p-umc1309	9	71.1	p-bnlg1401-1	10	145.3	p-bnlg2190
6	62.6	p-bnlg2243	7	126.0	p-umc1015	8	116.6	p-umc-2357				10	154.1	p-bnlg1360
6	74.1	p-bnlg1443	7	140.7	p-phi112	8	130.1	p-phi233376				10	183.0	p-phi041
6	115.6	p-umc1350	7	162.8	p-umc1695	8	198.1	bnlg1252						
			7	205.0	p-umc2333-1									

Supplementary Table S6 Position of different markers used in population 2

Chr.	position	locus	Chr.	position	locus	Chr.	position	locus	Chr.	position	locus	Chr.	position	locus
1	0	p-phi227562	2	0	bnlg1092-1	3	0	p-umc2071	4	0	p-bnlg490	5	0	bnlg653
1	26.1293	umc2064	2	8.2546	bnlg1092-2	3	14.5624	p-umc2105	4	174.0789	bnlg1755	5	39.5452	umc1153
1	26.1293	umc1064	2	17.7281	bnlg1297	3	49.7638	p-umc2050	4	195.7226	p-umc1117	5	95.3305	umc2136
1	48.1616	p-umc2025	2	74.7606	umc1736	3	91.4576	p-umc1174	4	210.3898	umc1142	5	130.2485	p-bnlg118
1	96.5526	p-bnlg1057	2	116.331	phi21				4	231.765	umc1945	5	160.6394	p-bnlg1046
1	138.6685	umc1568	2	152.9017	p-phi109642				4	261.5392	umc2285	5	189.912	umc1482
1	177.7792	umc1147	2	186.2758	umc2214				4	261.5392	bnlg1621	5	252.5327	umc1098
1	187.1235	p-bnlg1556	2	245.718	umc2019				4	261.5392	p-umc1101	5	346.2731	umc1226
			2	270.6567	bnlg1413				4	261.5392	p-umc2038	5	375.529	bnlg1287
			2	278.6734	bnlg1017				4	261.5392	p-bnlg2244	5	428.1743	p-umc2298
			2	301.757	umc0111				4	261.5392	p-bnlg2291	5	435.0837	p-umc2299
									4	283.948	umc1720	5	456.3255	bnlg565-1
									4	326.3287	umc1869	5	465.0242	bnlg565-2
									4	409.6965	umc1561	5	506.6375	umc1155
												5	560.2866	umc2292
												5	605.6332	umc1680
												5	608.8825	umc1792
												5	623.4851	bnlg143
**Chr.**	**position**	**locus**	**Chr.**	**position**	**locus**	**Chr.**	**position**	**locus**	**Chr.**	**position**	**locus**	**Chr.**	**position**	**locus**
6	0	p-umc2059	7	0	p-umc1695	8	0	P-BNLG162	9	0	umc1137	10	0	bnlg1450
6	21.9304	umc2324	7	48.4368	p-umc1015	8	38.4598	p-bnlg1194	9	46.5801	p-phi022	10	26.6584	umc1319
6	68.4021	phi077				8	51.3153	p-bnlg2235	9	89.5574	p-umc1982	10	61.0727	umc1152
6	117.559	p-umc1114				8	77.6363	p-phi080	9	117.5418	p-umc1231			
						8	81.2375	p-phi233376	9	127.5696	umc1078			
						8	100.9661	p-umc1309	9	171.5112	DUP029			
						8	127.8491	p-umc1663						
